# Metagenome from ACT-3/CF: an anaerobic chloroform-degrading microbial community

**DOI:** 10.1128/mra.00674-24

**Published:** 2024-09-19

**Authors:** Olivia Bulka, Elizabeth Edwards

**Affiliations:** 1 Department of Chemical Engineering and Applied Chemistry, University of Toronto, Toronto, Ontario, Canada; Montana State University, Bozeman, Montana, USA

**Keywords:** bioremediation, chloroform, microbial communities, metagenome

## Abstract

Here, we present the metagenome of an anaerobic chloroform respiring mixed microbial community used for bioremediation. Our objective was to obtain draft genomes of key microorganisms in the culture.

## ANNOUNCEMENT

Chloroform (CF) contaminates groundwater worldwide due to accumulation from many anthropogenic and natural sources, but through biostimulation or bioaugmentation, the power of microorganisms can be harnessed to degrade CF *in situ* (1–3). ACT-3 is an anaerobic enrichment culture originally derived from soil collected in 2001 from a site in Massachusetts [(42.4, –71.9); depth: 30′; pH: 7] contaminated with 1,1,1-trichloroethane (1,1,1-TCA) (4). ACT-3 was initially enriched on 1,1,1-TCA (4), but in 2008, a sub-transfer (ACT-3/CF) was instead provided CF (3), which has since been amended biweekly-monthly (~0.5 mM aqueous) alongside methanol/lactate/ethanol as electron donor. *Dehalobacter* sp. CF is the predominant strain; it respires CF to dichloromethane but relies on hydrogen, acetate, and undetermined additional nutrients supplied by a rich microbial community [>100 species (5)]. The *Dehalobacter* sp. CF genome was previously closed [SRX181448, CP003870 (6)], but much of the community remains obscure. This metagenomic data was assembled to provide insight into the microbes performing supporting roles in ACT-3/CF.

DNA from two 400-mL samples (one each in September and November 2020) was extracted using the Kingfisher Duo Prime MagMax microbiome kit (Thermo Scientific, Waltham, MA). The Genome Quebec Innovation Centre (Montréal, QC, Canada) prepared and sequenced both samples; the first used the NEBNext Ultra II DNA Library Prep Kit (New England BioLabs, Ipswich, MA) and the second used the SMRTbell Express Template Prep Kit 2.0 (Pacific Biosciences, Menlo Park, CA) without shearing or size selection. Illumina NovaSeq 6000 sequencing produced 88,022,042 paired-end reads (read length: 150 nt, average insert size: 250 nt), which were quality controlled with FastQC v0.11.9 (7). The first three low-quality bases were trimmed with Trimmomatic v0.39 (8). PacBio Sequel II sequencing produced 2,196,736 reads (*N*_50_: 64.5 kb). Illumina and PacBio reads were co-assembled with hybridSPAdes (SPAdes v3.15.0, python v3.6.12) (9), producing the 124.3 Mb ACT-3/CF metagenome (124,541 contigs, *N*_50_: 5.2 kb). All software used default parameters. Contigs > 1 kb were binned using MetaBAT2 v2.15, MaxBin v2.2.7, and CONCOCT v1.1.0 and were dereplicated with DAS Tool v1.1.2 (10–13) to select quality bins within Anvi’o v7 (14–16). Metagenome-assembled genomes (MAGs) were assigned taxonomy using GTDB R89.0 (14, 15). Coding sequences and RNA were detected with Prokka v1.14.6 (17). MAGs were manually curated using Anvi’o (14–16).

Eight high-quality (>90% complete, <5% redundant) and six medium-quality (>50% complete, <10% redundant) MAGs were recovered (Table 1), including one archaeon (*Methanosphaerula palustris*) and several bacterial phyla: Actinobacteriota (*n* = 1), Chloroflexota (*n* = 3), Desulfobacterota (*n* = 1), Spirochaetota (*n* = 2), Verrucomicrobiota (*n* = 1), and Firmicutes (*n* = 5, including *Dehalobacter* sp. CF). Among these organisms, several have been hypothesized to provide *Dehalobacter* sp. CF with cobamide—a critical cofactor for reductive dechlorination (18). After further analysis of their encoded metabolic potential, these MAGs will undoubtedly facilitate a deeper understanding of the interconnectedness of the ACT-3/CF microbial community and the resilience needed for bioremediation.

**TABLE 1:** Summary of metagenome-assembled genomes (MAGs) recovered from the ACT-3/CF metagenome assembly, including quality statistics and taxonomy

MAG	GTDB classification (R89.0)	Size (Gb)	No. contigs	*N*_50_ (Kb)	GC (%)	Quality	Completion (%)	Redundancy (%)	No. rRNA			No. tRNA	No. CDS
									5S	16S	23S		
metabat_bin_38	Archaea; Halobacteriota; Methanomicrobia; Methanomicrobiales; Methanosphaerulaceae; *Methanosphaerula palustris*	2.6	2	1999.7	58.6	high	94.7	3.9	6	3	3	54	2394
concoct_bin_8	Bacteria; Actinobacteriota; Actinomycetia; Propionibacteriales; Propionibacteriaceae; *Propionicimonas* sp002441465	3.0	1113	3.2	68.7	medium	88.7	4.2	0	0	1	38	3221
concoct_bin_52	Bacteria; Chloroflexota; Anaerolineae; Anaerolineales; Anaerolineaceae; 49–20; sp002436085	1.4	271	8.3	50.3	medium	63.4	1.4	0	0	0	18	1400
metabat_bin_4_sub	Bacteria; Chloroflexota; Anaerolineae; Anaerolineales; Anaerolineaceae; *Bellilinea* sp.	2.8	453	6.7	58.1	medium	76.1	2.8	0	0	0	27	2662
concoct_bin_22	Bacteria; Chloroflexota; Anaerolineae; Anaerolineales; Anaerolineaceae; UBA6107; sp003501835	3.2	8	2912.9	52.6	high	91.5	0.0	1	1	1	48	2896
concoct_bin_46	Bacteria; Desulfobacterota; Desulfovibrionia; Desulfovibrionales; Desulfovibrionaceae; *Solidesulfovibrio*	5.5	34	298.0	65.4	high	100.0	2.8	2	2	2	59	4826
concoct_bin_54_sub	Bacteria; Firmicutes; Clostridia; Christensenellales	3.6	45	172.3	52.3	high	97.2	1.4	1	1	1	46	3424
maxbin_019_sub	Bacteria; Firmicutes; Clostridia; Oscillospirales; Acutalibacteraceae; *Caproiciproducens* sp002359465	1.7	521	4.8	47.2	medium	50.7	9.9	0	0	0	12	1730
metabat_bin_31	Bacteria; Firmicutes; Clostridia; Peptostreptococcales; Anaerovoracaceae; UBA7709; sp002482405	4.6	76	92.9	51.6	high	94.4	2.8	1	1	1	38	3961
concoct_bin_20	Bacteria; Firmicutes; Desulfitobacteriia; Desulfitobacteriales; Syntrophobotulaceae; *Dehalobacter restrictus*	3.3	20	551.7	44.3	high	100	0	3	3	3	54	3081
metabat_bin_5	Bacteria; Firmicutes; Syntrophomonadia; Syntrophomonadales; Syntrophomonadaceae; *Syntrophomonas* sp002840245	3.5	59	77.8	46.9	medium	93.0	4.2	0	0	0	31	3304
concoct_bin_31_sub	Bacteria; Spirochaetota; Spirochaetia; Treponematales; UBA8932; UBA11090; sp002839205	3.3	97	66.1	62.6	high	90.1	1.4	2	2	2	44	2870
metabat_bin_19	Bacteria; Spirochaetota; Spirochaetia; Treponematales; UBA8932; UBA8932; sp002441395	3.2	1	3186.8	55.6	high	90.1	2.8	2	2	2	49	2889
concoct_bin_7_sub	Bacteria; Verrucomicrobiota; Verrucomicrobiae; Pedosphaerales; Pedosphaeraceae; UBA6082; sp002428665	5.4	106	88.7	58.6	medium	93.0	8.5	0	1	1	52	4438

## Data Availability

All reads and assemblies are available on NCBI under BioProject PRJNA80805. This Whole Genome Shotgun project is deposited under the GenBank accession no. JAVKYP000000000. The version described in this paper is JAVKYP010000000. Illumina and PacBio reads are deposited under Sequence Read Archive accession nos. SRX21661671 and SRX21661672, respectively. All MAGs described here are available at 10.6084/m9.figshare.26053156 (fasta) and 10.6084/m9.figshare.26058811 (annotations).
